# Characterization of the tumor-infiltrating immune repertoire in muscle invasive bladder cancer

**DOI:** 10.3389/fimmu.2023.986598

**Published:** 2023-02-03

**Authors:** Raquel Benítez, Katherine Yu, Marina Sirota, Núria Malats, Silvia Pineda

**Affiliations:** ^1^ Genetic and Molecular Epidemiology Group, Spanish National Cancer Research Centre (CNIO) and CIBERONC, Madrid, Spain; ^2^ Bakar Computational Health Sciences Institute, University of California, San Francisco (UCSF), San Francisco, CA, United States; ^3^ Department of Statistics and Data Science, Complutense University of Madrid (UCM), Madrid, Spain

**Keywords:** B-cell repertoire, T-cell repertoire, subtyping, tumor microenevironment, muscle invasive bladder cancer (MIBC), tumor infiltration

## Abstract

**Introduction:**

Muscle-invasive bladder cancer (MIBC) is a heterogeneous disease with several taxonomic molecular subtypes showing different genetic, clinical, and epidemiological profiles. It has been suggested that MIBC-subtypes follow different tumorigenesis pathways playing decisive roles at different stages of tumor development, resulting in distinct tumor microenvironment containing both innate and adaptive immune cells (T and B lymphocytes). We aim to characterize the MIBC tumor microenvironment by analyzing the tumor-infiltrating B and T cell repertoire according to the taxonomic molecular subtypes.

**Methods:**

RNAseq data from 396 MIBC samples included in TCGA were considered. The subtype information was collected from the international consensus taxonomic classification describing six subtypes: Basal/Squamous-like (Ba/Sq), Luminal papillary (LumP), Luminal non-Specify (LumNS), Luminal unstable (LumU), Stroma-rich, and Neuroendocrine-like (NE-like). Using MiXCR, we mapped the RNA read sequences to their respective B-cell receptor (BCR) and T-cell receptor (TCR) clonotypes. To evaluate the BCR and TCR differences among subtypes, we compared diversity measures (richness and diversity) using a Wilcoxon test and we performed a network analysis to characterize the clonal expansion. For the survival analysis stratified by subtypes, Cox regression models adjusted for age, region, and pathological stage were performed.

**Results:**

Overall, we found different patterns of tumor-infiltrating immune repertoire among the different MIBC subtypes. Stroma-rich and Ba/Sq tumors showed the highest BCR and TCR infiltration while LumP showed the lowest. In addition, we observed that the Ba/Sq and Stroma-rich tumors were more clonally expanded than the Luminal subtypes. Moreover, higher TCR richness and diversity were significantly associated with better survival in the Stroma-rich and Ba/Sq subtypes.

**Discussion:**

This study provides evidence that MIBC subtypes present differences in the tumor microenvironment, in particular, the Ba/Sq and the Stroma-rich are related with a higher tumoral-infiltrating immune repertoire, which seems to be translated into better survival. Determining the causes of the different tumoral-infiltrating immune repertoire according to the MIBC molecular subtypes will help to improve our understanding of the disease and the distinct responses to immunotherapy of MIBC.

## Introduction

Bladder cancer (BC) is the fourth most common cancer in Northern America and Europe among men and its incidence is still rising (1, 2)⁠. Urothelial bladder cancer (UBC) morphology represents 95% of BC. Overall, UBC is considered an immunogenic tumor due to its relatively high tumor mutational burden (3)⁠ and its responsiveness to Bacillus Calmette–Guerin (BCG) bladder instillations and checkpoint inhibitors (4)⁠. However, not all patients benefit from these therapies, possibly, because BC is not a single disease.

Most of UBC (80%) are diagnosed as non-muscle invasive tumors. While this is a milder form of UBC, a large proportion (40%) of patients suffer of multiple recurrences with some of them invading the detrusor muscle (MIBC), this being a life-threat event requiring a more aggressive treatment (5, 6)⁠. MIBC has further been classified according to somatic DNA-based and RNA-based features. Regarding the latter, several taxonomic classifications with different numbers and names of MIBC subtypes have been proposed (7–17). Recently, Kamoun et al. published an international consensus paper based on a network-based analysis done with 1750 MIBC transcriptomic profiles from six independent MIBC studies. The authors reported up to six subtypes: Luminal papillary (LumP), Luminal unstable (LumU), Luminal Non-Specified (LumNS), Stroma-rich, Basal/Squamous-like (Ba/Sq), and Neuroendocrine-like) (17). Interestingly, it has been observed that each of the subtypes has distinct differentiation patterns, oncogenic mechanisms, tumor microenvironments, as well as histological and clinical associations.

The network of immunoregulatory pathways in bladder cancer is quite complex, with several immune mechanisms arresting the effective antitumor T-cell response. T cells and dendritic cells (DCs) expressed inhibitory receptors in their membranes, repressing tumor growth. Type 1 T helper (TH1) cells favor the generation of an anti-tumor immune response. However, type 2 T helper (TH2) cells favor pro-tumor immune responses. Other immune cell types that promote tumor development are myeloid-derived suppressor cells (MDSCs), M2 macrophages and regulatory T (Treg) cells. In addition, Mast cells have been implicated in an indirect pro-tumor role, although the mechanism remains unclear (18).

This immune infiltration varies according to bladder cancer stages. Thus, non-muscle invasive bladder cancer (NMIBC) and MIBC show significant differences in the infiltration of immune cells. Furthermore, Kamoun et al. characterized the tumor microenvironment for the different MIBC subtypes using cell deconvolution tools and they observed that the Ba/Sq and the Stroma-rich subtypes had higher immune and stromal infiltration as well as distinct immune cell populations than the rest of subtypes. Even though the immune infiltration was mainly found within these two subtypes, it showed distinct immune cell populations. Ba/Sq tumors were enriched in cytotoxic lymphocytes and natural killer cells, whereas stroma-rich tumors overexpressed T- and B-cell markers. LumNS tumors were the only luminal type associated with immune infiltration signals; these were mainly for B and T lymphocytes (17). These differences could help in the selection of the patients for immunotherapies. (19)

In UBC, there are extensive evidences for an overall suppression of immunosurveillance responses within the tumor. However, little is known about the antigen specific responses (20). Among all the immune cell populations, B and T cells are key components of the adaptive immune response. T cells are involved in cell-mediated immunity, whereas B cells are primarily responsible for antibody responses against the specific antigens recognition through the B cell receptors (BCR) or immunoglobulins (Ig) Both receptors can recognize a large number of molecules. The BCR and TCR loci are form by recombining a set of variable (V), diversity (D) and joining (J) gene segments and its diversity is mainly concentrated in the complementary-determining region 3 (CDR3).

The BCR are made up of two heavy chains (IGH) and two light chains, the kappa (κ) chains (IGK) and the lambda (λ) chains (IGL). The receptor diversification arises from two different processes: somatic recombination and somatic hypermutation (21)⁠. By contrast, T cell receptors (TCR) are either TCRαβ or TCRγδ. Approximately 95% of T cells express a TCRαβ receptor, consisting of a TCRα (TRA) and a TCRβ (TRB) chain. The remaining 5% are made by a TCRγ (TRD) and a TCRδ (TRG) chain. These TCR chains are highly diverse in their variable domains (22)⁠.

The immune cell infiltration harboring these receptors may play decisive roles at different stages of tumor development resulting in a tumor microenvironment containing different balances of T and B cell receptors, in addition to the cancer cells and surrounding stroma. Their impact on tumor progression and treatment response has been suggested. In fact, T-cell infiltration play a central role in modern immunotherapy response in bladder cancer, among other cancers (4, 23, 24)⁠, whereas the role of B-cell infiltration has yet to be defined (25)⁠. Furthermore, this infiltration could be used as immunological biomarkers which may drive towards patient stratification (26).

Thus, given the fact the MIBC subtypes presented different immune microenvironment and clinical behavior, we hypothesized that the adaptive immune infiltration of these receptors is also distinct across the MIBC subtypes. Therefore, our aim was to further characterize the MIBC immune microenvironment by analyzing the tumor-infiltrating B- and T- cell repertoire according to the tumor taxonomic molecular subtypes towards a better understanding of MIBC progression pathways.

## Material and methods

### TCGA data

The study population included 404 MIBCs patients from TCGA with available consensus taxonomic subtype data by Kamoun et al. (17)⁠ based on RNAseq tumor data. Information on tumor gene expression (RNA-seq), demographic, and clinicopathological characteristics were retrieved through TCGA data portal (https://tcga-data.nci.nih.gov/tcga/). Mutational rate data was retrieved from Thorsson et al. (27)⁠. All of the patients provided informed consent to TCGA. Subtype information was directly extracted from the original published paper (17)⁠. The final number of patients analyzed was 396 (LumP=126, LumNS=20, LumU=53, Stroma-rich=45, and Ba/Sq=152). We excluded 6 neuroendocrine-like tumors because of the insufficient number for the posterior subtype analysis. Two patients were also discarded based on the out-ranged and very low values presented in the TCR reads (**Table S1**).

### BCR and TCR data extraction

B-cell receptors (BCR) and T-cell receptors (TCR) were extracted from the RNAseq FASTQ files using the bioinformatic software MiXCR (28)⁠. We applied the pipeline described in https://mixcr.readthedocs.io/en/master/ for alignments using paired-end RNA-seq. MiXCR captures all CDRs and framework regions of immune genes and permits the assembly of full-length clonotypes. In this paper, we extracted BCR and TCR and defined the clonotypes according to their CDR3 sequences retrieved from the bulk RNA-seq data. The median number of reads and clones for the four datasets are displayed in **Table S2**. TRD and TRG reads and clones were very few, therefore we decided to filter out these receptors for the analysis.

### Richness and diversity analyses

To evaluate the number of BCR/TCR clones and their frequency we assessed the richness and the diversity. They were calculated through Expression and Entropy measurements, respectively (29)⁠. The number of BCR/TCR reads can be highly dependent on the sequencing depth. Therefore, we accounted for this by calculating the expression dividing the number of reads by the total number of sequenced reads in the RNA-seq FASTQ files. Expression was estimated with the following formula:


$$Ig_{i}/TR_{i}=\frac{M_{i}}{N_{i}+M_{i}};i=1,\text{...},N$$

Where M_i_ is the number of reads that map to a specific VDJ recombination and N_i_ is the number of reads that map to a anything else in the genome in n samples.

In addition, to take into consideration not only the number of clones but its frequency, Shannon entropy (H index) was estimated as:


$$H=-\sum_{i=1}^{N}p_{i}\log_{2}(p_{i});i=1,\text{...},N$$

Where N is the number of unique clones and p_i_ is the frequency of clone i. We defined a clone as those reads that had the same V and J gene, same CDR3 length, and 90% of nucleotide identity for BCR, and 95% for TCR. We restricted this analysis to those reads that estimated the CDR3 region.

### Network analysis

The network used to assess the overall clonal nature and the dominance of a clone was generated applying an algorithm similar to that already described in previous publications (29, 30)⁠. Briefly, each vertex represents a B-cell or T-cell sequence where the size indicates the number of identical chains. An edge (defined by the clone definition: same V and J segments, same CDR3 length, and 90%/95% nucleotide identity between CDR3s for BCR/TCR, respectively) between two vertex indicates that the sequence belongs to the same clone and clusters define each clone in the repertoire. The analysis was done using igraph package in R using the layout with_graphopt option to generate the plot.

Then, the network was quantified calculating the Gini Index for vertex size (clonal expansion) and cluster size (clonal diversification). Gini Index is a measure of unevenness extensively used to measure wealth distribution. A Gini coefficient of zero expresses perfect equality and a Gini coefficient of 1 expressed maximal inequality. It measures the inequality among values of frequency distribution. We used the Gini function from ineq package in R to calculate the Gini coefficient for vertex size and cluster size distribution. When applied to vertex size, the overall clonal nature is represented. If it was closer to 1, vertices were unequal, showing expansion of some of them, and closer to 0, otherwise. When applied to cluster size, clonal dominance was represented. If closer to 1, clusters were unequal and therefore represented dominant clones; if closer to 0, all clusters were of equal size. Finally, all these information was considered together to compare the clonal expansion and diversification trends by subtypes.

### Statistical analysis

To evaluate the BCR and TCR differences among subtypes, we compared diversity measures (expression and entropy) using a Wilcoxon rank test. We also checked the correlation between the clinic-pathological variables available in TCGA and diversity measures for all receptors (IGH, IGK, IGL, TRA and TRB) stratifying by MIBC subtype and using Wilcoxon rank test when the variable was categorical and Spearman correlation test when continuous. In order to assess the correlation by MIBC subtypes between the mutational rates (silent and non-silent mutation rates, SMR and NSMR, respectively) and the diversity measures, a Spearman correlation test was applied.

The inflammatory score was calculated based on Thorsson et al. (27)⁠ scores calculation approach. The authors applied CIBERSORT to the TCGA RNA-seq data to estimate the relative fraction of 22 immune cell types within the leukocyte compartment. More specifically, they aggregate the cell types of interest to generate the different scores. According to this, we calculated the inflammatory score by adding the relative fraction of inflammation related immune cells (Inflammatory score = Monocytes + Macrophages.M0 + Macrophages.M1 + Macrophages.M2 + Dendritic.cells.resting + Dendritic.cells.activated + Mast.cells.resting + Mast.cells.activated + Neutrophils + Eosinophils + B.cells.naive + B.cells.memory + T.cells.CD4.naive + T.cells.CD4.memory.resting + T.cells.CD4.memory.activated + T.cells.follicular.helper + T.cells.regulatory + T.cells.gamma.delta + T.cells.CD8 + T.helper + NK.cells.resting + NK.cells.activated). To check the correlation between the inflammatory score and both measures, richness and diversity for all receptors (IGH, IGK, IGL, TRA and TRB) stratified by subtype, a Spearman correlation test was used.

Survival analysis were performed to assess the association between the diversity measures (expression and entropy) and overall survival (OS). Hazard ratios and 95% confidence intervals were estimated with Cox regression models adjusted for age, region, and pathological disease stage. The different BCR and TCR measurements were included in the models to evaluate their prognostic value. Cox model considering the potential interaction between the BCR and TCR measurements and the subtypes were also calculated.

## Results

Sociodemographic features were similar across subtypes, the LumNS and LumU being slightly older than the rest of the subtypes (**Table 1**). As expected, the Stroma-rich and Ba/Sq subtypes had less papillary features. The LumNS subtype was the most advanced at diagnosis (65% of the tumors in Stage IV). The summary of the sequenced reads by MIBC subtypes is showed in **Table S2**. For all of MIBC subtypes, the number of reads was higher for the BCR compared to the TCR clonotypes. Stroma-rich subtype (mean(sd) = 41954 [2290 – 1312355]) and LumNS (mean(sd) = 24998 [2156 - 304394]) were the subtypes with the highest number of BCR reads, whereas the highest amount of TCR reads was shown by the Ba/Sq (mean(sd) = 404.5 [13 - 3975]) and Stoma-rich (mean(sd) = 308 [65 - 5753]) subtypes (**Table S2**).

**Table 1:** Sociodemographic and clinical features for MIBC cases according to subtypes.

	All cases	LumP	LumNS	LumU	Stroma-rich	Ba/Sq	p.value
	N=396	N=126	N=20	N=53	N=45	N=152	
**Age at diagnosis:**							0.024
<=60	104 (26.3%)	45 (36.0%)	3 (15.0%)	13 (24.5%)	6 (13.3%)	37 (24.3%)	
61-70	121 (30.6%)	38 (30.4%)	5 (25.0%)	11 (20.8%)	18 (40.0%)	49 (32.2%)	
>70	170 (43.0%)	42 (33.6%)	12 (60.0%)	29 (54.7%)	21 (46.7%)	66 (43.4%)	
**Sex:**							0.274
Female	104 (26.3%)	32 (25.4%)	7 (35.0%)	8 (15.1%)	12 (26.7%)	45 (29.6%)	
Male	292 (73.7%)	94 (74.6%)	13 (65.0%)	45 (84.9%)	33 (73.3%)	107 (70.4%)	
**Region:**							0.86
USA+Canada	334 (84.6%)	103 (82.4%)	18 (90.0%)	46 (86.8%)	40 (88.9%)	127 (83.6%)	
Europe	43 (10.9%)	16 (12.8%)	2 (10.0%)	3 (5.66%)	4 (8.89%)	18 (11.8%)	
Brazil+Puerto Rico	18 (4.56%)	6 (4.80%)	0 (0.00%)	4 (7.55%)	1 (2.22%)	7 (4.61%)	
**BMI:**							0.05
<25	148 (42.4%)	60 (53.1%)	5 (27.8%)	17 (34.7%)	17 (44.7%)	49 (37.4%)	
>=25	201 (57.6%)	53 (46.9%)	13 (72.2%)	32 (65.3%)	21 (55.3%)	82 (62.6%)	
**Smoking status:**							0.631
Non-smoker	107 (27.9%)	41 (33.1%)	5 (27.8%)	13 (25.0%)	10 (23.3%)	38 (26.0%)	
Ever smoker	276 (72.1%)	83 (66.9%)	13 (72.2%)	39 (75.0%)	33 (76.7%)	108 (74.0%)	
**Histology**:							<0.001
Papillary	132 (33.8%)	73 (58.4%)	7 (35.0%)	16 (30.8%)	8 (17.8%)	28 (18.8%)	
Non-Papillary	259 (66.2%)	52 (41.6%)	13 (65.0%)	36 (69.2%)	37 (82.2%)	121 (81.2%)	
**Disease Stage:**							<0.001
STAGE I-II	127 (32.2%)	70 (56.5%)	1 (5.00%)	14 (26.4%)	4 (8.89%)	38 (25.0%)	
STAGE III	136 (34.5%)	30 (24.2%)	6 (30.0%)	20 (37.7%)	17 (37.8%)	63 (41.4%)	
STAGE IV	131 (33.2%)	24 (19.4%)	13 (65.0%)	19 (35.8%)	24 (53.3%)	51 (33.6%)	

### BCR/TCR infiltration is significantly different across MIBC subtypes

**Figure 1:**
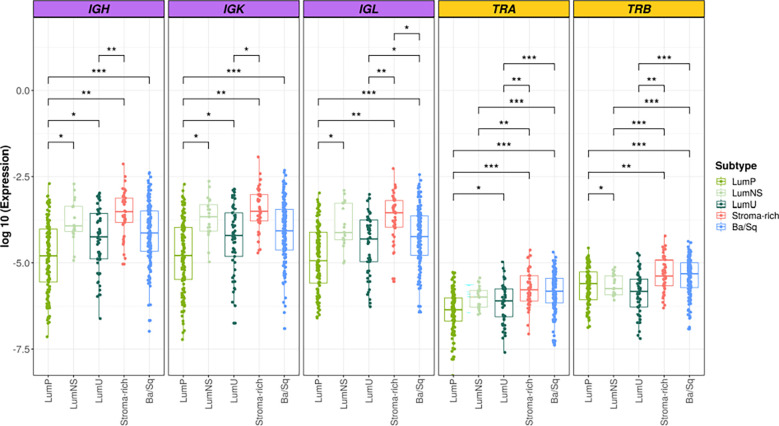
BCR and TCR richness among the different MIBC subtypes. BCR related results are plotted in purple and TCR in yellow. In the Y axis the logarithm of the expression is represented. Each box of the boxplots is a subtype (see legend). The differences between richness across the subtypes are displayed only when significant. * → 0.05 > p > 0.01; ** → 0.01 > p > 0.001; *** → p < 0.001.

**Figure 2:**
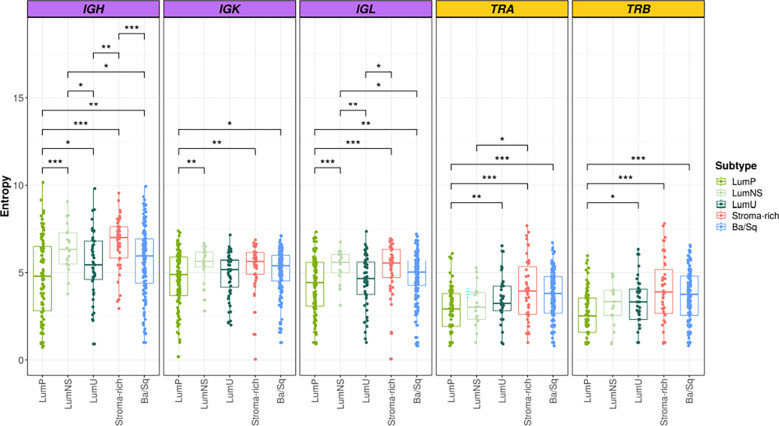
BCR and TCR diversity among the different MIBC subtypes. BCR related results are plotted in purple and TCR in yellow. In the Y axis entropy is represented. Each box of the boxplots is a subtype (see legend). The differences between diversity across the subtypes are displayed only when significant. Wilcoxon test: * → 0.05 > p > 0.01; ** → 0.01 > p > 0.001; *** → p < 0.001.

Overall, we found significant differences of BCR and TCR richness and diversity across MIBC subtypes (**Figures 1**, **2**). The highest BCR infiltration was observed for the Stroma-rich subtype that, jointly with Ba/Sq tumors, showed the highest TCR richness and diversity, too. On the other hand, LumP tumors presented the lowest BCR and TCR infiltration pattern. Wilcoxon rank test results comparing the TCR and BCR diversity measures between the different subtypes are reported in **Tables S3, S4**.

**Figure 3:**
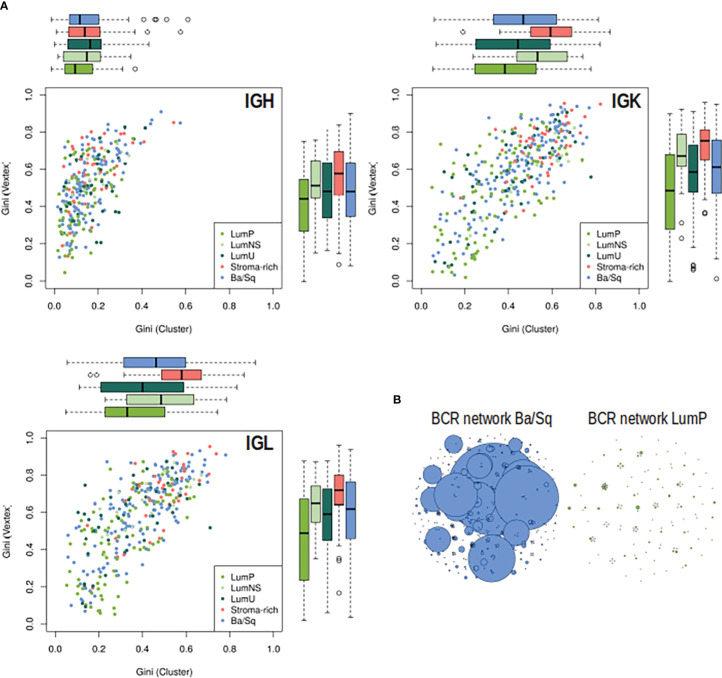
BCR clonal expansion and diversification by subtypes. **(A)** On the Y axis, clonal expansion is represented by using the Gini Index and on the X axis, clonal diversification is displayed by Gini Vertex. The plot is colored by subtype, each dot is a MIBC sample and the boxplot represented the distribution of each clonal measurement by subtype. **(B)** B-cell repertoire networks from two samples representing one Ba/Sq (blue) and one LumP (green). Each vertex represents a unique BCR being the vertex size defined by the number of identical BCRs considering the nucleotide sequences. An edge exists between vertices when they belong to the same clone as defined before, so clusters are groups of interconnected vertices forming a clone.

Significant differences in BCR clonal expansion and clonal diversification between the different subtypes were also found through the network analyses (**Figure 3**; **Tables S5, S6**). The Stroma-rich subtype showed the highest BCR clonal expansion levels while the LumP showed the lowest. The rest of the subtypes behaved similar in terms of BCR clonal expansion. The Stroma-rich subtype showed the highest levels of dominant clones for IGL and IGK and LumP showed the lowest. Higher levels of IGH dominant clones were observed for the LumNS subtype and the lowest levels were observed for the LumP, again.

### Association between mutational rates and BCR/TCR infiltration varies across the different MIBC subtypes

**Figure 4:**
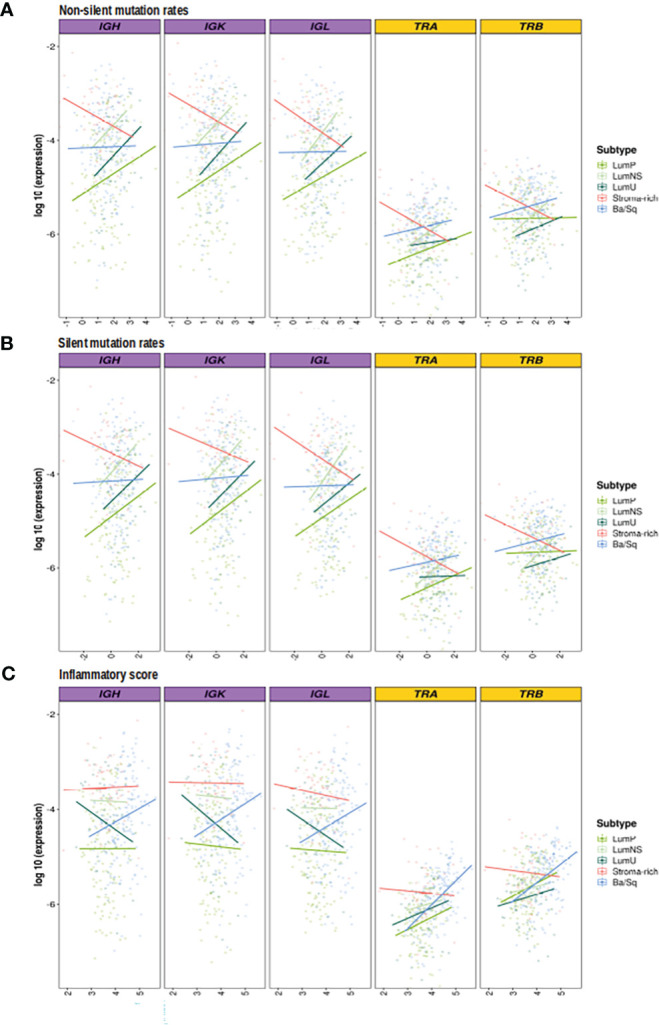
Correlation between mutational rates and inflammatory score with richness by subtypes. BCR related results are plotted in purple and TCR in yellow. In the Y axis the logarithm of the expression is displayed. On the X axes, the logarithm 10 of the **(A)** non-silent **(B)** silent mutational rates, **(C)** inflammation score are plotted. Each line, is the regression line assessed in the correlation test performed by subtypes and they are colored by them.

While no correlation was observed between the mutational rates and the infiltration measures overall, the correlation patterns were highly heterogeneous across the different MIBC subtypes (**Figures 4A, B**, **S1A, B**; **Tables 2**, **3**). In the Stroma-rich subtype, mutational rates showed a negative correlation trend with both BCR and TCR richness (NSMR rho: IGH=-0.24, IGK=-0.28, IGL=-0.27, TRA=-0.26, TRB=-0.24; SMR rho: IGH=-0.25, IGK=-0.29, IGL=-0.29, TRA=-0.30, TRB=-0.28) and TCR diversity (NSMR rho: TRA=-0.12, TRB=-0.26; SMR rho: TRA=-0.17, TRB=-0.31). A slightly positive correlation between the mutational rates and the TCR richness (NSMR rho: TRA=0.18, p.value=0.03; TRB=0.19, p.value=0.02; SMR rho: TRA=0.16, p.value=0.05; TRB=0.16, p.value=0.05) and diversity (NSMR rho: TRA=0.18, p.value=0.03; TRB=0.21 p.value=0.01; SMR rho: TRA=0.17, p.value=0.04; TRB=0.18 p.value=0.03) was found in the Ba/Sq. In addition, LumP showed a positive correlation with both BCR and TCR richness (NSMR rho: IGH=0.21, p.value=0.02; IGK=0.22, p.value=0.01; IGL=0.21, p.value=0.02; TRA=0.23, p.value=9.7e-03; SMR rho: IGH=0.19, p.value=0.03; IGK=0.20, p.value=0.02; IGL=0.20, p.value=0.03; TRA=0.22, p.value=0.01) and with TRA diversity (NSMR rho: 0.30, p.value=0.02; SMR rho: 0.28, p.value=4.1e-03) (**Tables 2**, **3**).

**Table 2:** Correlation between the two mutational rates and inflammatory score and richness for all receptors by subtypes.

Richness	Subtype	All		LumP		LumNS		LumU		Stroma-rich		Ba/Sq	
	Chain Type	R	p-value	R	p-value	R	p-value	R	p-value	R	p-value	R	p-value
Non-silent mutation rate (NSMR)													
	IGH	0.09	8.01e-02	0.21	1.88e-02	0.33	0.158	0.29	3.67e-02	-0.24	0.116	0.02	0.818
	IGK	0.09	6.26e-02	0.22	1.30e-02	0.40	7.92e-02	0.28	4.57e-02	-0.28	6.19e-02	0.03	0.748
	IGL	0.08	0.116	0.21	1.72e-02	0.46	4.27e-02	0.28	4.15e-02	-0.27	7.20e-02	0.01	0.867
	TRA	0.11	2.26e-02	0.23	9.66e-03	0.47	3.84e-02	0.06	0.670	-0.26	8.99e-02	0.18	3.00e-02
	TRB	0.03	0.542	0.01	0.880	0.64	3.03e-03	0.14	0.310	-0.24	0.116	0.19	2.06e-02
Silent mutation rate (SMR)													
	IGH	0.07	0.149	0.19	3.01e-02	0.30	0.192	0.25	6.97e-02	-0.25	9.53e-02	0.02	0.844
	IGK	0.08	0.127	0.20	2.33e-02	0.38	9.88e-02	0.24	8.61e-02	-0.29	5.40e-02	0.02	0.784
	IGL	0.07	0.192	0.20	2.58e-02	0.43	6.07e-02	0.25	7.24e-02	-0.29	5.64e-02	0.01	0.859
	TRA	0.09	6.11e-02	0.22	1.49e-02	0.52	2.06e-02	0.04	0.769	-0.30	4.75e-02	0.16	5.17e-02
	TRB	0.01	0.800	0.02	0.809	0.63	3.58e-03	0.11	0.439	-0.28	6.22e-02	0.16	5.28e-02
Inflammatory score													
	IGH	0.11	2.82e-02	0.03	0.716	-0.04	0.888	-0.25	7.57e-02	-0.10	0.508	0.20	1.61e-02
	IGK	0.11	3.51e-02	0.01	0.884	-0.07	0.781	-0.26	6.24e-02	-0.15	0.310	0.22	8.17e-03
	IGL	0.11	3.58e-02	0.00	0.962	-0.15	0.541	-0.27	5.79e-02	-0.14	0.348	0.21	9.73e-03
	TRA	0.44	8.54e-20	0.29	1.19e-03	0.15	0.551	0.21	0.136	-0.03	0.862	0.47	2.40e-09
	TRB	0.35	1.56e-12	0.28	1.58e-03	0.06	0.815	0.18	0.196	-0.06	0.684	0.36	7.41e-06

Spearman correlation test was used.

**Table 3:** Correlation between the two mutational rates and inflammatory score and diversity for all receptors by subtypes.

Diversity	Subtype	All		LumP		LumNS		LumU		Stroma-rich		Ba/Sq	
	Chain Type	R	p-value	R	p-value	R	p-value	R	p-value	R	p-value	R	p-value
Non-silent mutation rate (NSMR)													
	IGH	0.07	0.200	0.18	6.50e-02	0.48	3.33e-02	0.07	0.622	-0.06	0.700	0.01	0.865
	IGK	0.01	0.868	0.06	0.498	0.5	2.71e-02	-0.01	0.956	0.15	0.330	-0.1	0.212
	IGL	0.03	0.494	0.08	0.358	0.37	0.109	0.05	0.706	-0.08	0.583	0.03	0.751
	TRA	0.15	5.00e-03	0.3	2.21e-03	0.32	0.163	0.07	0.667	-0.12	0.452	0.18	2.80e-02
	TRB	0.08	0.114	0.13	0.191	0.47	3.79e-02	0.02	0.872	-0.26	8.46e-02	0.21	1.29e-02
Silent mutation rate (SMR)													
	IGH	0.06	0.249	0.18	5.98e-02	0.46	4.27e-02	0.05	0.727	-0.06	0.706	0.01	0.882
	IGK	0	0.925	0.03	0.767	0.5	2.61e-02	-0.04	0.791	0.19	0.210	-0.11	0.193
	IGL	0.03	0.618	0.07	0.427	0.36	0.122	0.02	0.906	-0.1	0.510	0.03	0.719
	TRA	0.12	1.83e-02	0.28	4.08e-03	0.36	0.118	0.05	0.733	-0.17	0.262	0.17	4.34e-02
	TRB	0.07	0.206	0.12	0.227	0.5	2.47e-02	0.03	0.831	-0.31	4.12e-02	0.18	2.74e-02
Inflammatory score													
	IGH	0.06	0.265	0.05	0.619	-0.06	0.815	-0.17	0.246	-0.15	0.324	0.1	0.252
	IGK	-0.05	0.374	-0.13	0.156	0.1	0.678	-0.19	0.172	-0.32	3.39e-02	-0.01	0.873
	IGL	-0.04	0.467	-0.11	0.254	-0.09	0.705	-0.05	0.710	-0.15	0.324	-0.08	0.338
	TRA	0.39	2.46e-14	0.33	6.14e-04	0.24	0.329	0.16	0.299	-0.04	0.806	0.43	9.58e-08
	TRB	0.4	2.04e-15	0.37	9.11e-05	0.31	0.197	0.19	0.180	0.04	0.777	0.4	1.43e-06

Spearman correlation test was used.

### Ba/Sq BCR/TCR infiltration is significantly associated with inflammatory score

Ba/Sq subtype showed a significant positive correlation with both BCR and TCR richness (IGH: rho=0.20, p.value=1.6e-02; IGK: rho=0.22, p.value=8.2e-03; IGL: rho=0.21, p.value=9.7e-03, TRA: rho=0.47, p.value=2.4e-09; TRB: rho=0.36, p.value=7.4e-06) and TCR diversity (TRA: rho=0.43, p.value=9.6e-08; TRB: rho=0.40, p.value=1.4e-06). In addition, a significant positive correlation between the inflammatory score and TCR richness (TRA: rho=0.29, p.value=1.2e-03; TRB: rho=0.28, p.value=1.6e-03) and diversity (TRA: rho=0.33, p.value=6.1e-06; TRB: rho=0.37, p.value=9.1e-05) was observed for the LumP subtype (**Figures 4C**, **S1C**; **Tables 2**, **3**). Interestingly, BCR and TCR richness and diversity did not show any pattern of correlation with the inflammatory score in the Stroma-rich subtype.

### There is no association between the clinical data and BCR/TCR infiltration

We explored the association between the clinic-pathological variables available in TCGA and both measures, richness and diversity, for all receptors (IGH, IGK, IGL, TRA and TRB) by stratifying by subtype. No associations for any of the clinical variables was observed (**Table S7**).

### BCR and TCR infiltration is associated with overall survival in Ba/Sq and Stroma-rich subtypes

**Figure 5:**
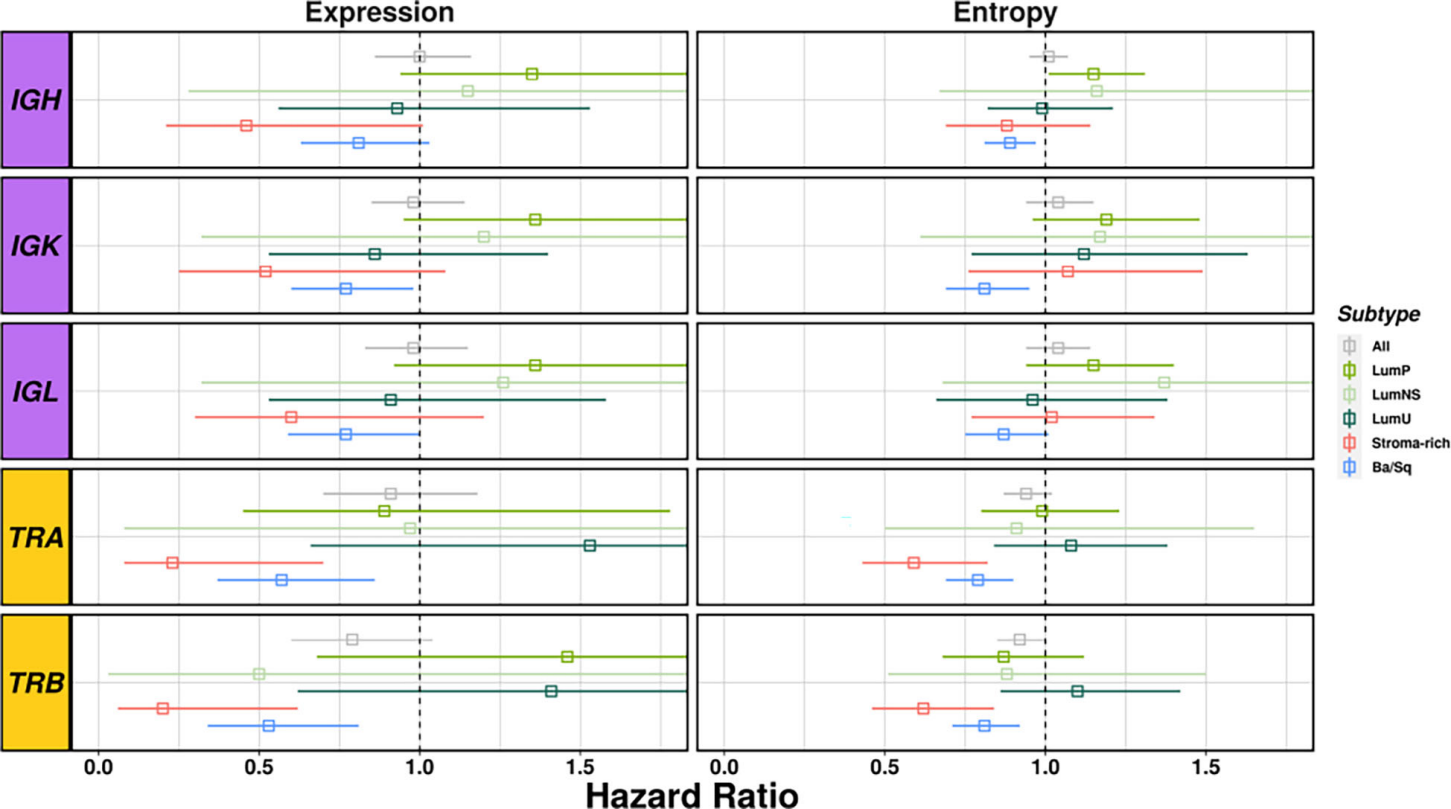
Survival analyses considering richness and diversity for all receptors results by subtype. BCR related results are plotted in purple and TCR in yellow. Each square represents the Hazard Ratio and its corresponding lines the 95%CI. The different colors indicates the different subtypes (see legend) and all cases together are display in gray.

Richness and diversity were associated with OS when we stratified by subtypes (**Table S8** and **Figure 5**) showing an interaction effect between the BCR and TCR measurements with the different subtypes (**Table S9**). Ba/Sq subtype showed significantly better OS for higher TCR richness (HR[95% CI]: TRA=0.57 [0.37-0.86], TRB=0.53 [0.34-0.81]) and diversity (HR[95% CI]: TRA=0.79 [0.69-0.9], TRB=0.81 [0.71-0.92]) and BCR richness (HR[95% CI]: IGH=0.81 [0.63-1.03], IGK=0.77 [0.6-0.98], IGL=0.77 [0.59-1.00]), and Stroma-rich subtype showed significantly better OS for higher TCR richness (HR[95% CI]: TRA=0.23 [0.08-0.7], TRB=0.2 [0.06-0.62]) and diversity (HR[95% CI]: TRA=0.59 [0.43-0.82], TRB=0.62 [0.46-0.84]). Interestingly, OS was not associated with diversity measures if stratification by subtypes was not performed. BCR and TCR infiltration were not significantly associated with OS in either of Luminal subtypes.

## Discussion

Cancer classifications, such as the pathological, the molecular, and the taxonomic, aim to improve the patient management. Molecular subtyping studies have allowed the allocation of cancer into homogeneous groups that are considered to harbor similar molecular and clinical characteristics. Furthermore, this has helped researchers to identify both actionable targets for drug design as well as biomarkers for response prediction. In deep, molecular subtyping studies have allowed to better correlate cancer cases with clinical outcomes than the traditional classifications of cancer (31–33)⁠. MIBC is a heterogeneous disease with several taxonomic molecular subtypes showing different genetic, clinical, and epidemiological profiles (17, 34)⁠. It has also been suggested that MIBC-subtypes follow different tumorigenesis pathways playing decisive roles at different stages of tumor development and resulting in a tumor microenvironment containing different balances of adaptive immune cells (T and B lymphocytes) (17)⁠. In addition, the different MIBC subtypes have been associated with different therapeutics options (16). However, despite the growing evidences of subtyping clinical implications, MIBC subtypes have yet to enter into routine clinical practice (35).

While a very recent study detailed the UBC immune-profile, it did not considered the different molecular subtypes (19). To our knowledge, this is the first study characterizing the MIBC immune microenvironment by analyzing the tumor-infiltrating B and T repertoire according to the taxonomic molecular subtypes using RNAseq data from 396 MIBC samples included in TCGA. Furthermore, we report on the association of tumor-infiltrating immune repertoire with mutational rates, inflammatory score, overall survival, and clinico-pathological features, across the different MIBC subtypes.

We observed large differences on BCR and TCR richness and diversity, as well as on BCR clonal expansion and diversification among the different MIBC subtypes. Stroma-rich and Ba/Sq tumors showed the highest BCR and TCR infiltration while LumP subtype showed the lowest. In addition, we observed that the Ba/Sq and Stroma-rich tumors were more clonally expanded than the Luminal subtypes. Moreover, the correlation between mutational rates and inflammatory score with BCR/TCR measurements highly varied across subtypes.

A high infiltration in Ba/Sq tumors is further supported by the observation that Schistosoma-associated bladder cancer arises from a chronic granulomatous inflammation and irritation leading to squamous cell carcinoma subtype of the bladder (36)⁠ Urinary tract infections (UTIs) have been controversially established as risk factor for bladder cancer (37)⁠. Since it has been shown that during UTIs an adaptive immune response is generated (38)⁠, we could think that subtyping bladder cancer could help to better establish their relationship with bladder cancer.

The high infiltration of BCR and TCR in the Stroma- rich subtypes relies on the fact that these tumors displayed overexpression of smooth muscle, myofibroblast, fibroblast and endothelial gene signatures, intermediate urothelial differentiation and overexpression of B-cell markers (17, 39). The fact that molecular subtyping is performed on biopsy specimens representing only a fraction of the tumor mass, warrants particular caution when considering this subtype. While some tumors are actually stroma-rich, some biopsy specimens are stroma-rich due to chance or sampling at the tumor margin (40). Hence, it is necessary to be careful in drawing conclusions about this subtype.

Nevertheless, the fact that immune infiltration is associated with mutational rate in Stroma-rich tumors and with the inflammatory score in Ba/Sq subtype suggest that the source and type of immune infiltration may be different in these two subtypes. Our observation is also supported by Kamoun et al. that reported distinct immune cell populations in these two MIBC subtypes (17)⁠. Specifically, we found that the tumor-infiltrating immune repertoire inversely correlates with the mutational rates in the Stroma-rich subtype. This inverse correlation could be explained because this subtype is characterized by a high stromal infiltration, mainly of smooth cells, fibroblasts, and myofribroblasts that could cover the cancer cells where the mutations mainly originate (17)⁠.

In general, high mutational rates lead to the formation of tumor neoantigens, making tumors more immunogenic and more sensitive to immunotherapy (41)⁠. The above observations, jointly with the fact that higher TCR richness and diversity was associated with better survival only in the Stroma-rich and the Ba/Sq subtypes, reinforce the need to further explore the joint role of the tumor subtype and the immune infiltration in the anti-tumor response (42)⁠.

The three luminal subtypes showed the lowest BCR and TCR infiltration. This group of subtypes is characterized by a less aggressive presentation of the disease and a better prognosis (11, 43). Moreover, the immune infiltration patterns positively correlated with the mutational rates. Intriguingly, the correlation was only observed for the BCR richness and diversity, but not for the TCR measurements. Whether the type of mutations in the luminal subtypes are more immunogenic than those in the other subtypes requires further exploration.

The impact of the immune infiltration pattern on prognosis varies across the different subtypes. While BASQ-like subtype has been characterized by a more aggressive presentation of the disease and worse prognosis (11, 44)⁠, we were further able to differentiate a Ba/Sq tumors subset with better prognosis associated with TCR infiltration. Similar results were observed for Stroma-rich tumors, characterized by a better survival. The fact that BCR and TCR richness and diversity were not associated with OS in the luminal subtypes may explain why these tumors are poor responders to immunotherapy (45, 46)⁠, a fact that could be used for patient stratification in the clinics.

There are some limitations that should be considered when interpreting these results. First, we applied MiXCR tool to map the read sequences using RNAseq data to their respective BCR and TCR clonotypes as done elsewhere (29, 42)⁠. In this line, we have previously explored the tumor infiltrating B cell repertoires across tumor types (42)⁠ showing a large variability on BCR infiltration across tumor types and an increase clonality in primary tumors compared to adjacent non-tumor tissues. Despite these tools provide accurate annotations, further studies with targeted sequencing are necessary to validate the extracted features and associations. Another limitation of this study was the limited clinical data available in TCGA since the consortium’s main objective was the detail molecular characterization of the tumors. This impaired dawning clear conclusions from the lack of association between the tumor-infiltrating immune repertoire and clinic-pathological conditions. Hence, additional analyses in independent studies with more completed data are needed. We are also aware that some of the subtypes extracted from the MIBC samples had limited sample size (LumNS=20, NE-like=6). Indeed, NE-like tumors were excluded from the analysis due to this reason. Therefore, these findings will need to be further validated in a larger sample sized study. In addition, the characterization of the risk factors associated with the different subtypes would strongly increase the clinical and treatment potential significance of the findings.

This study provides sound evidence that MIBC subtypes present differences in the tumor B- and T-cell immune repertoire. In particular, the Stroma-rich and Ba/Sq tumors are related with a higher tumoral-infiltrating immune repertoire, however the origin of the immune infiltration may be different in these two subtypes. Interestingly, the Ba/Sq subtype immune infiltration correlated with inflammation-related cells infiltrating the tumor while the Stroma-rich immune infiltration correlated with the mutational rates. Importantly, BCR and TCR infiltration was associated with a better overall survival in both Ba/Sq and Stroma-rich subtypes. A better definition of the different immune-related mechanism leading to several MIBC taxonomic subtypes will improve our understanding of the disease and the identification of novel therapeutic strategies.

## Data availability statement

The original contributions presented in the study are included in the article/**Supplementary Material**. Further inquiries can be directed to the corresponding authors.

## Ethics statement

The studies involving human participants were reviewed and approved by TCGA data. The patients/participants provided their written informed consent to participate in this study.

## Author contributions

SP, NM, and RB conceived the study design and analysis plan. RB performed the data analysis. KY, SP, and RB extracted the data with MiXCR and helped in the data analysis. SP, NM, and MS supervised the work. All authors contributed to the article and approved the submitted version.
