# Supplementary material for: Characterization of the tumor-infiltrating immune repertoire in muscle invasive bladder cancer
Source: Front Immunol. 2023 Feb 3;14:986598. doi: 10.3389/fimmu.2023.986598 (PMC9936234; doi:10.3389/fimmu.2023.986598)

Supplementary Material

Supplementary Tables

Table S1. Population exclusion criteria.
Table S2. Sequenced reads summary.
Table S3. BCR and TCR richness differences among subtypes.
Table S4. BCR and TCR diversity differences among subtypes.
Table S5. BCR and TCR clonal expansion differences among subtypes.
Table S6. BCR and TCR clonal diversification differences among subtypes.
Table S7. Association between clinic-pathological variables and richness and diversity for all receptors by subtypes.
Table S8. Survival analyses results stratified by subtype.
Table S9. Survival analyses results by subtype.

Supplementary Figures

Figure S1. Survival analyses considering richness and diversity for all receptors results by subtype.

Supplementary Table 1. Population exclusion criteria.

**Total Population:**	404 MIBC patients with available subtype information
**Excluded:**	6 NE-like patients
	2 very low TCR reads patients (out-range values)
**Final Population:**	396 MIBC patients

Supplementary Table 2. Sequenced reads summary. Median [minimum – maximum] number of reads and clones for the B-cell receptors (BCR) and T-cell receptors (TCR) extracted from the RNAseq FASTQ files using MiXCR.

Subtype	N	Total reads	BCR reads	BCR clones	TCR reads	TCR clones
LumP	126	59973904.5; [24352465 - 125701334]	3007; [34 - 273248]	125.5; [0 - 5006]	199; [12 - 2066]	6.5; [0 - 161]
LumNS	20	63378782.5; [28831877 - 84723910]	24998; [2156 - 304394]	535; [107 - 3424]	167; [37 - 734]	19.5; [2 - 75]
LumU	53	60513511; [39453636 - 98818357]	10147; [75 - 208053]	312; [3 - 3688]	136; [9 - 2651]	16; [0 - 226]
Stroma-rich	45	58286305; [30694936 - 127022287]	41954; [2290 - 1312355]	929; [29 - 4890]	308; [65 - 5753]	31; [1 - 526]
Ba/Sq	152	59959506; [8408416 - 149204452]	13427.5; [59 - 783231]	354; [3 - 5261]	404.5; [13 - 3975]	29.5; [0 - 276]

Supplementary Table 3. BCR and TCR richness differences among subtypes. Wilcoxon rank test results.

Chain type	Group 1	Group 2	n1	n2	Statistic	df	p-val
IGH	LumP	LumNS	126	20	-2.25	20	**3.60e-02**
		LumU	126	53	-2.19	82	**3.10e-02**
		Stroma-rich	126	45	-3.28	45	**2.00e-03**
		Ba/Sq	126	152	-4.01	198	**8.74e-05**
	LumNS	LumU	20	53	1.30	24	0.207
		Stroma-rich	20	45	-1.61	63	0.113
		Ba/Sq	20	152	0.20	29	0.845
	LumU	Stroma-rich	53	45	-2.67	49	**1.00e-02**
		Ba/Sq	53	152	-1.92	190	5.60e-02
	Stroma-rich	Ba/Sq	45	152	1.94	52	5.80e-02
IGK	LumP	LumNS	126	20	-2.32	20	**3.10e-02**
		LumU	126	53	-2.37	71	**2.00e-02**
		Stroma-rich	126	45	-2.95	45	**5.00e-03**
		Ba/Sq	126	152	-4.13	192	**5.34e-05**
	LumNS	LumU	20	53	1.17	26	0.252
		Stroma-rich	20	45	-1.67	59	0.101
		Ba/Sq	20	152	0.26	28	0.793
	LumU	Stroma-rich	53	45	-2.40	48	**2.00e-02**
		Ba/Sq	53	152	-1.56	170	0.122
	Stroma-rich	Ba/Sq	45	152	1.94	48	5.90e-02
IGL	LumP	LumNS	126	20	-2.49	20	**2.20e-02**
		LumU	126	53	-1.76	77	8.20e-02
		Stroma-rich	126	45	-3.37	45	**2.00e-03**
		Ba/Sq	126	152	-4.00	188	**9.05e-05**
	LumNS	LumU	20	53	1.73	24	9.70e-02
		Stroma-rich	20	45	-1.53	63	0.132
		Ba/Sq	20	152	0.43	29	0.670
	LumU	Stroma-rich	53	45	-2.89	48	**6.00e-03**
		Ba/Sq	53	152	-2.27	194	**2.40e-02**
	Stroma-rich	Ba/Sq	45	152	2.01	52	**4.90e-02**
TRA	LumP	LumNS	126	20	-1.83	27	7.80e-02
		LumU	126	53	-2.12	65	**3.80e-02**
		Stroma-rich	126	45	-3.66	45	**6.64e-04**
		Ba/Sq	126	152	-6.93	193	**6.15e-11**
	LumNS	LumU	20	53	-0.49	66	0.622
		Stroma-rich	20	45	-2.97	51	**4.00e-03**
		Ba/Sq	20	152	-4.27	83	**5.22e-05**
	LumU	Stroma-rich	53	45	-2.70	56	**9.00e-03**
		Ba/Sq	53	152	-3.38	143	**9.38e-04**
	Stroma-rich	Ba/Sq	45	152	1.14	54	0.261
TRB	LumP	LumNS	126	20	2.02	54	**4.90e-02**
		LumU	126	53	1.53	110	0.130
		Stroma-rich	126	45	-2.76	49	**8.00e-03**
		Ba/Sq	126	152	-4.17	257	**4.11e-05**
	LumNS	LumU	20	53	-0.27	64	0.789
		Stroma-rich	20	45	-3.43	50	**1.00e-03**
		Ba/Sq	20	152	-5.63	100	**1.68e-07**
	LumU	Stroma-rich	53	45	-3.28	53	**2.00e-03**
		Ba/Sq	53	152	-4.99	166	**1.52e-06**
	Stroma-rich	Ba/Sq	45	152	1.06	55	0.295

n1 & n2: sample size group 1 and 2 respectively; df: degrees of freedom.

Supplementary Table 4. BCR and TCR diversity differences among subtypes. Wilcoxon rank test results.

Chain type	Group 1	Group 2	n1	n2	Statistic	df	p-val
IGH	LumP	LumNS	97	20	-4.34	46	**7.57e-05**
		LumU	97	47	-2.06	112	**4.10e-02**
		Stroma-rich	97	45	-5.66	122	**1.02e-07**
		Ba/Sq	97	143	-3.15	183	**2.00e-03**
	LumNS	LumU	20	47	2.28	49	**2.70e-02**
		Stroma-rich	20	45	-0.58	42	0.567
		Ba/Sq	20	143	2.19	32	**3.60e-02**
	LumU	Stroma-rich	47	45	-3.20	89	**2.00e-03**
		Ba/Sq	47	143	-0.54	83	0.593
	Stroma-rich	Ba/Sq	45	143	3.40	92	**1.00e-03**
IGK	LumP	LumNS	118	20	-2.84	35	**7.00e-03**
		LumU	118	52	-0.95	117	0.344
		Stroma-rich	118	45	-2.64	91	**1.00e-02**
		Ba/Sq	118	151	-2.54	219	**1.20e-02**
	LumNS	LumU	20	52	1.92	42	6.20e-02
		Stroma-rich	20	45	0.42	46	0.675
		Ba/Sq	20	151	1.33	26	0.195
	LumU	Stroma-rich	52	45	-1.62	91	0.109
		Ba/Sq	52	151	-1.11	85	0.272
	Stroma-rich	Ba/Sq	45	151	0.93	67	0.355
IGL	LumP	LumNS	116	20	-4.29	44	**9.44e-05**
		LumU	116	53	-1.03	112	0.307
		Stroma-rich	116	45	-3.58	88	**5.62e-04**
		Ba/Sq	116	149	-3.03	211	**3.00e-03**
	LumNS	LumU	20	53	2.90	56	**5.00e-03**
		Stroma-rich	20	45	0.43	57	0.671
		Ba/Sq	20	149	2.33	30	**2.70e-02**
	LumU	Stroma-rich	53	45	-2.34	93	**2.10e-02**
		Ba/Sq	53	149	-1.31	81	0.193
	Stroma-rich	Ba/Sq	45	149	1.65	65	0.104
TRA	LumP	LumNS	83	19	-1.05	29	0.303
		LumU	83	38	-2.72	70	**8.00e-03**
		Stroma-rich	83	43	-3.87	65	**2.58e-04**
		Ba/Sq	83	141	-4.82	189	**2.98e-06**
	LumNS	LumU	19	38	-1.11	41	0.272
		Stroma-rich	19	43	-2.28	51	**2.70e-02**
		Ba/Sq	19	141	-1.96	26	6.00e-02
	LumU	Stroma-rich	38	43	-1.41	77	0.162
		Ba/Sq	38	141	-0.79	63	0.433
	Stroma-rich	Ba/Sq	43	141	0.99	59	0.325
TRB	LumP	LumNS	89	19	-1.90	27	6.90e-02
		LumU	89	39	-2.56	68	**1.30e-02**
		Stroma-rich	89	43	-4.21	62	**8.32e-05**
		Ba/Sq	89	138	-5.66	203	**5.22e-08**
	LumNS	LumU	19	39	-0.16	39	0.872
		Stroma-rich	19	43	-1.81	50	7.60e-02
		Ba/Sq	19	138	-1.39	25	0.177
	LumU	Stroma-rich	39	43	-1.87	77	6.50e-02
		Ba/Sq	39	138	-1.49	64	0.141
	Stroma-rich	Ba/Sq	43	138	0.97	58	0.337

n1 & n2: sample size group 1 and 2 respectively; df: degrees of freedom.

Supplementary Table 5. BCR and TCR clonal expansion differences among subtypes. Wilcoxon rank test results.

Chain type	Group 1	Group 2	n1	n2	Statistic	df	p-val
IGH	LumP	LumNS	84	20	-2.65	30	**1.30e-02**
		LumU	84	44	-2.72	92	**8.00e-03**
		Stroma-rich	84	45	-5.14	96	**1.42e-06**
		Ba/Sq	84	136	-2.98	182	**3.00e-03**
	LumNS	LumU	20	44	0.56	37	0.582
		Stroma-rich	20	45	-1.08	36	0.289
		Ba/Sq	20	136	0.91	26	0.372
	LumU	Stroma-rich	44	45	-2.09	87	**4.00e-02**
		Ba/Sq	44	136	0.40	80	0.691
	Stroma-rich	Ba/Sq	45	136	2.93	84	**4.00e-03**
IGK	LumP	LumNS	110	20	-4.69	35	**4.15e-05**
		LumU	110	50	-3.07	101	**3.00e-03**
		Stroma-rich	110	45	-7.88	119	**1.70e-12**
		Ba/Sq	110	147	-4.93	215	**1.64e-06**
	LumNS	LumU	20	50	1.78	46	8.10e-02
		Stroma-rich	20	45	-1.22	35	0.231
		Ba/Sq	20	147	1.60	27	0.120
	LumU	Stroma-rich	50	45	-3.59	89	**5.45e-04**
		Ba/Sq	50	147	-0.59	80	0.555
	Stroma-rich	Ba/Sq	45	147	4.09	92	**9.04e-05**
IGL	LumP	LumNS	103	20	-5.19	47	**4.40e-06**
		LumU	103	51	-2.63	110	**1.00e-02**
		Stroma-rich	103	45	-6.85	115	**3.75e-10**
		Ba/Sq	103	147	-4.46	207	**1.33e-05**
	LumNS	LumU	20	51	2.29	56	**2.60e-02**
		Stroma-rich	20	45	-1.04	46	0.301
		Ba/Sq	20	147	1.85	35	**7.30e-02**
	LumU	Stroma-rich	51	45	-3.50	93	**7.28e-04**
		Ba/Sq	51	147	-0.92	89	0.360
	Stroma-rich	Ba/Sq	45	147	3.37	92	**1.00e-03**
TRA	LumP	LumNS	45	15	-0.06	28	0.951
		LumU	45	29	0.09	62	0.926
		Stroma-rich	45	37	2.02	75	**4.80e-02**
		Ba/Sq	45	110	-2.72	93	**8.00e-03**
	LumNS	LumU	15	29	0.13	32	0.894
		Stroma-rich	15	37	1.59	21	0.126
		Ba/Sq	15	110	-2.01	22	**5.70e-02**
	LumU	Stroma-rich	29	37	1.68	46	**1.00e-01**
		Ba/Sq	29	110	-2.52	51	**1.50e-02**
	Stroma-rich	Ba/Sq	37	110	-5.77	114	**7.00e-08**
TRB	LumP	LumNS	43	13	0.35	31	0.729
		LumU	43	22	-1.57	37	0.126
		Stroma-rich	43	31	0.37	56	0.715
		Ba/Sq	43	111	-4.41	106	**2.50e-05**
	LumNS	LumU	13	22	-1.75	33	**8.90e-02**
		Stroma-rich	13	31	0.05	39	0.958
		Ba/Sq	13	111	-4.33	27	**1.81e-04**
	LumU	Stroma-rich	22	31	1.67	46	0.101
		Ba/Sq	22	111	-1.50	34	0.143
	Stroma-rich	Ba/Sq	31	111	-3.85	54	**3.19e-04**

n1 & n2: sample size group 1 and 2 respectively; df: degrees of freedom.

Supplementary Table 6. BCR and TCR clonal diversification differences among subtypes. Wilcoxon rank test results.

Chain type	Group 1	Group 2	n1	n2	Statistic	df	p-val
IGH	LumP	LumNS	66	19	-1.08	26	0.288
		LumU	66	41	-2.06	73	**4.30e-02**
		Stroma-rich	66	44	-1.97	77	5.20e-02
		Ba/Sq	66	120	-1.89	160	6.00e-02
	LumNS	LumU	19	41	-0.46	37	0.651
		Stroma-rich	19	44	-0.40	37	0.690
		Ba/Sq	19	120	0.00	25	1.000
	LumU	Stroma-rich	41	44	0.06	83	0.949
		Ba/Sq	41	120	0.67	71	0.505
	Stroma-rich	Ba/Sq	44	120	0.59	75	0.557
IGK	LumP	LumNS	101	20	-4.49	32	**8.92e-05**
		LumU	101	51	-1.59	93	0.116
		Stroma-rich	101	44	-8.02	113	**1.07e-12**
		Ba/Sq	101	145	-3.82	215	**1.76e-04**
	LumNS	LumU	20	51	2.69	47	**1.00e-02**
		Stroma-rich	20	44	-1.18	33	0.247
		Ba/Sq	20	145	2.17	28	**3.90e-02**
	LumU	Stroma-rich	51	44	-4.73	87	**8.67e-06**
		Ba/Sq	51	145	-1.18	81	0.242
	Stroma-rich	Ba/Sq	44	145	5.01	99	2.35e-06
IGL	LumP	LumNS	91	20	-2.67	29	**1.20e-02**
		LumU	91	48	-1.19	89	0.238
		Stroma-rich	91	44	-6.58	100	**2.23e-09**
		Ba/Sq	91	144	-3.11	201	**2.00e-03**
	LumNS	LumU	20	48	1.56	40	0.126
		Stroma-rich	20	44	-1.76	33	8.70e-02
		Ba/Sq	20	144	0.91	26	0.370
	LumU	Stroma-rich	48	44	-4.26	88	**5.09e-05**
		Ba/Sq	48	144	-1.13	80	0.262
	Stroma-rich	Ba/Sq	44	144	4.22	90	**5.77e-05**
TRA	LumP	LumNS	6	3	-1.20	2	0.334
		LumU	6	7	-0.50	10	0.625
		Stroma-rich	6	8	2.03	8	7.70e-02
		Ba/Sq	6	46	-0.75	9	0.471
	LumNS	LumU	3	7	0.85	3	0.457
		Stroma-rich	3	8	1.98	2	0.177
		Ba/Sq	3	46	0.95	2	0.434
	LumU	Stroma-rich	7	8	1.94	8	9.00e-02
		Ba/Sq	7	46	0.04	8	0.972
	Stroma-rich	Ba/Sq	8	46	-3.82	28	**6.91e-04**
TRB	LumP	LumNS	9	4	0.84	9	0.421
		LumU	9	9	0.47	16	0.643
		Stroma-rich	9	10	2.10	13	5.60e-02
		Ba/Sq	9	48	0.13	12	0.898
	LumNS	LumU	4	9	-0.38	9	0.714
		Stroma-rich	4	10	1.09	5	0.323
		Ba/Sq	4	48	-0.94	5	0.395
	LumU	Stroma-rich	9	10	1.55	13	0.143
		Ba/Sq	9	48	-0.47	13	0.649
	Stroma-rich	Ba/Sq	10	48	-2.93	24	**7.00e-03**

n1 & n2: sample size group 1 and 2 respectively; df: degrees of freedom.

Supplementary Table 7. Association between clinico-pathological variables and richness and diversity for all receptors by subtypes. Wilcoxon rank test was used when the variable was categorical and Spearman correlation test when continuous. Empty cells are found when data to perform the analysis was missing

Variable	Group 1	Group 2	Chain Type	Richness						Diversity					
**Age**				All	LumP	LumNS	LumU	Stroma-rich	Ba/Sq	All	LumP	LumNS	LumU	Stroma-rich	Ba/Sq
	-	-	IGH	4.53e-02	0.352	0.747	0.549	0.788	0.777	0.304	0.280	0.508	0.613	0.412	0.943
			IGK	1.89e-02	0.181	0.820	0.495	0.962	0.740	0.460	0.797	0.297	0.493	0.781	0.330
			IGL	6.65e-02	0.327	0.474	0.364	0.869	0.986	0.530	0.661	0.798	0.228	0.333	0.228
			TRA	0.389	0.349	0.398	0.125	0.368	0.367	0.336	0.416	0.152	0.083	0.561	0.582
			TRB	0.454	0.407	0.869	0.190	0.899	0.993	0.170	0.085	0.480	0.214	0.149	0.298
**Sex**															
	Male	Female	IGH	0.462	0.248	0.747	0.850	0.664	0.466	5.00e-03	0.028	0.378	0.760	0.741	0.088
			IGK	0.492	0.157	0.774	0.707	0.486	0.367	7.00e-03	0.145	0.378	0.784	0.132	0.081
			IGL	0.355	0.112	0.718	0.613	0.686	0.479	7.00e-03	0.068	0.245	0.678	0.674	0.085
			TRA	0.537	0.467	0.824	0.632	0.452	0.546	0.273	0.605	0.691	0.833	0.342	0.280
			TRB	0.666	0.454	0.478	0.808	0.187	3.40e-02	0.150	0.895	0.859	0.631	0.780	0.130
**BMI**															
	-	-	IGH	0.143	0.503	0.370	0.901	0.521	0.679	0.500	0.656	0.160	0.935	0.156	0.887
			IGK	0.186	0.467	0.530	0.967	0.364	0.782	0.983	0.895	0.093	0.739	0.061	0.850
			IGL	0.125	0.492	0.152	0.847	0.332	0.550	0.581	0.368	0.116	0.865	0.549	0.487
			TRA	0.111	0.374	0.107	0.779	0.499	0.896	7.11e-02	0.254	0.115	0.899	0.708	0.707
			TRB	0.843	0.870	8.08e-02	0.534	0.885	0.469	0.216	0.810	0.627	0.744	0.645	0.409
**Smoking status**															
	Non-smoker	Ever smoker	IGH	0.278	0.513	0.291	0.310	0.203	0.888	0.436	0.167	0.149	0.091	0.054	0.129
			IGK	0.268	0.441	0.266	0.547	0.258	0.853	5.70e-02	0.091	0.236	0.023	0.576	0.022
			IGL	0.286	0.608	0.368	7.00e-02	0.238	0.616	1.20e-02	0.021	0.018	0.113	0.017	0.061
			TRA	0.897	0.911	0.718	6.90e-02	0.283	0.632	0.796	0.895	0.913	0.064	0.261	0.662
			TRB	0.717	0.193	0.999	4.30e-02	0.284	0.388	0.902	0.611	0.296	0.157	0.580	0.171
**Histology**															
	Papillary	Non-Papillary	IGH	2.20e-02	0.109	0.364	0.456	0.788	0.952	1.00e-03	0.014	0.356	0.335	0.893	0.870
			IGK	1.80e-02	4.40e-02	0.308	0.712	0.623	0.889	4.00e-03	0.006	0.923	0.959	0.938	0.682
			IGL	3.00e-03	5.10e-02	0.532	0.381	0.832	0.435	3.40e-02	0.010	0.154	0.463	0.611	0.076
			TRA	7.56e-04	0.116	0.890	0.667	0.641	0.225	2.10e-05	0.117	0.405	0.834	0.668	0.117
			TRB	0.523	0.300	0.700	0.941	0.788	0.437	4.15e-04	0.177	0.255	0.388	0.948	0.496
**Disease stage**															
	STAGE I-II	STAGE III	IGH	0.115	0.216		0.812	0.423	0.739	0.242	0.561		0.461	0.525	0.773
			IGK	0.157	0.289		0.834	0.365	0.977	0.723	0.728		0.833	0.879	0.665

			IGL	0.135	0.517		0.950	0.287	0.960	0.414	0.710		0.360	0.334	0.840
			TRA	0.234	7.90e-03		8.92e-02	0.885	0.742	0.897	0.094		0.306	0.656	0.418
			TRB	0.841	0.495		7.93e-02	0.876	0.691	0.986	0.114		0.247	0.549	0.571
	STAGE I-II	STAGE IV	IGH	0.170	0.227		0.835	0.616	0.779	3.00e-03	0.137		0.866	0.476	0.842
			IGK	0.164	0.163		0.845	0.525	0.639	2.20e-02	0.174		0.795	0.596	0.401
			IGL	0.182	0.182		0.671	0.380	0.610	9.00e-03	0.308		0.982	0.268	0.831
			TRA	0.275	0.502		0.160	0.534	1.26e-02	0.542	0.723		0.212	0.673	0.003
			TRB	2.90e-02	1.77e-03		0.282	0.411	4.36e-02	0.455	0.573		0.444	0.478	0.030
	STAGE III	STAGE IV	IGH	0.649	0.965		0.972	0.583	0.460	6.80e-02	0.086		0.368	0.866	0.945
			IGK	0.686	0.801		0.984	0.551	0.558	4.60e-02	0.147		0.586	0.465	0.593
			IGL	0.632	0.608		0.697	0.528	0.418	5.80e-02	0.224		0.337	0.550	0.977
			TRA	3.20e-02	0.258		0.562	0.291	3.42e-03	0.638	0.359		0.708	0.928	0.026
			TRB	4.10e-02	4.52e-02		0.364	0.274	8.25e-02	0.454	0.483		0.687	0.898	0.082
**TUR**															
	yes	no	IGH	0.283	0.205	0.528	0.552	0.732	0.953	0.413	0.414	0.126	0.907	0.444	0.826
			IGK	0.271	0.181	0.491	0.394	0.977	0.757	0.824	0.964	0.042	0.999	0.089	0.480
			IGL	0.342	0.165	0.565	0.649	0.826	0.725	0.802	0.567	0.118	0.559	0.815	0.503
			TRA	6.20e-02	0.227	0.728	0.308	0.522	0.789	0.203	0.320	0.669	0.234	0.575	0.957
			TRB	0.445	0.506	0.772	0.239	0.904	0.393	0.377	0.293	0.935	0.161	0.492	0.692
**BCG**															
	no	yes	IGH	0.800	0.804		1.01e-04	0.453	1.36e-03	0.548	0.494		0.419	0.376	0.833
			IGK	0.643	0.637		4.03e-04	0.440	9.82e-04	0.433	0.143		0.829	0.680	0.327
			IGL	0.729	0.933		1.27e-03	0.457	2.94e-02	0.904	0.360		0.508	0.035	0.510
			TRA	0.164	0.958		3.10e-03	0.968	0.104	8.50e-02	0.735		0.227	0.358	0.068
			TRB	0.108	0.944		3.57e-03	0.967	3.53e-02	0.478	0.500		0.110	0.485	0.212
**Cystectomy**															
	yes	no	IGH	0.113	0.187	0.528	0.552	0.298	0.841	0.383	0.207	0.126	0.907	0.762	0.497
			IGK	0.113	0.177	0.491	0.394	9.10e-02	0.667	0.842	0.697	0.042	0.999	0.000	0.180
			IGL	0.242	0.134	0.565	0.649	0.824	0.668	0.781	0.852	0.118	0.559	0.976	0.967
			TRA	2.20e-02	5.10e-02	0.728	0.308	2.80e-02	0.843	7.30e-02	0.145	0.669	0.234	0.494	0.995
			TRB	0.285	0.554	0.772	0.239	0.515	0.478	0.185	0.199	0.935	0.161	0.851	0.787
**Chemotherapy**															
	yes	no	IGH	0.260	0.644	0.524	0.195	0.182	0.257	1.30e-02	0.251	0.733	0.490	0.763	0.165
			IGK	0.243	0.631	0.601	0.147	0.164	0.193	0.163	0.102	0.820	0.434	0.241	0.455
			IGL	0.242	0.653	0.691	7.50e-02	0.177	0.370	4.60e-02	0.341	0.664	0.179	0.659	0.619
			TRA	0.545	0.505	0.762	0.882	0.938	0.455	0.146	0.101	0.639	0.371	0.844	0.579
			TRB	0.844	0.614	0.785	0.924	0.490	0.220	6.00e-02	0.188	0.591	0.833	0.962	0.105

**Radiotherapy**													
	yes	no	IGH	0.812	4.11e-05	0.552	0.828	0.757	0.244	0.068	0.907	0.330	0.771
			IGK	0.926	1.42e-05	0.394	0.776	0.877	0.505	0.364	0.999	0.434	0.320
			IGL	0.955	2.59e-06	0.649	0.817	0.792	0.436	0.478	0.559	0.411	0.774
			TRA	0.556	3.61e-07	0.308	0.704	0.464	0.269	0.014	0.234	0.524	0.675
			TRB	0.240	0.695	0.239	0.588	0.537	0.994	0.070	0.161	0.245	0.935

Table S8. Survival analyses results stratified by subtype. Cox models stratified by subtype results adjusted by age, gender, stage and centre.

Model considered	All N=396		LumP N=126		LumNS N=20		LumU N=53		Stroma-rich N=45		Ba/Sq N=152	
**Richness**												
log_{10}(expression)												
IGH	1 [0.86-1.16]	0.979	1.35 [0.94-1.95]	0.107	1.15 [0.28-4.75]	0.845	0.93 [0.56-1.53]	0.765	0.46 [0.21-1.01]	5.30e-02	0.81 [0.63-1.03]	8.86e-02
IGK	0.98 [0.85-1.14]	0.821	1.36 [0.95-1.95]	9.31e-02	1.2 [0.32-4.48]	0.781	0.86 [0.53-1.4]	0.548	0.52 [0.25-1.08]	8.14e-02	0.77 [0.6-0.98]	3.72e-02
IGL	0.98 [0.83-1.15]	0.814	1.36 [0.92-2.01]	0.121	1.26 [0.32-4.9]	0.740	0.91 [0.53-1.58]	0.743	0.6 [0.3-1.2]	0.152	0.77 [0.59-1.00]	4.61e-02
TRA	0.91 [0.7-1.18]	0.464	0.89 [0.45-1.78]	0.744	0.97 [0.08-12.18]	0.979	1.53 [0.66-3.54]	0.318	0.23 [0.08-0.7]	9.75e-03	0.57 [0.37-0.86]	7.82e-03
TRB	0.79 [0.6-1.04]	9.61e-02	1.46 [0.68-3.12]	0.331	0.5 [0.03-7.73]	0.618	1.41 [0.62-3.21]	0.408	0.2 [0.06-0.62]	5.66e-03	0.53 [0.34-0.81]	3.16e-03
**Diversity**												
entropy												
IGH	1.01 [0.95-1.07]	0.854	1.15 [1.01-1.31]	3.59e-02	1.16 [0.67-2.01]	0.592	0.99 [0.82-1.21]	0.955	0.88 [0.69-1.14]	0.335	0.89 [0.81-0.97]	8.99e-03
IGK	1.04 [0.94-1.15]	0.451	1.19 [0.96-1.48]	0.111	1.17 [0.61-2.25]	0.640	1.12 [0.77-1.63]	0.547	1.07 [0.76-1.49]	0.713	0.81 [0.69-0.95]	9.18e-03
IGL	1.04 [0.94-1.14]	0.469	1.15 [0.94-1.4]	0.162	1.37 [0.68-2.78]	0.382	0.96 [0.66-1.38]	0.810	1.02 [0.77-1.34]	0.912	0.87 [0.75-1.01]	6.49e-02
TRA	0.94 [0.87-1.02]	0.122	0.99 [0.8-1.23]	0.941	0.91 [0.5-1.65]	0.750	1.08 [0.84-1.38]	0.547	0.59 [0.43-0.82]	1.51e-03	0.79 [0.69-0.9]	5.28e-04
TRB	0.92 [0.85-1]	5.51e-02	0.87 [0.68-1.12]	0.270	0.88 [0.51-1.5]	0.638	1.1 [0.86-1.42]	0.450	0.62 [0.46-0.84]	1.84e-03	0.81 [0.71-0.92]	1.67e-03

Table S9. Survival analyses results by subtype. Cox models (Overall Survival ~ Subtype*Chain type measure) results adjusted by age, gender, stage and centre.

Ref: Ba/Sq	**LumP**		**LumNS**		**LumU**		**Stroma-rich**	
Model considered	N=126		N=20		N=53		N=45	
**Richness**								
log_{10}(expression)								
IGH	1.19 [0.99-1.42]	6.95e-02	0.89 [0.55-1.46]	0.649	1.07 [0.85-1.34]	0.590	0.83 [0.6-1.13]	0.238
IGK	1.22 [1.01-1.46]	3.42e-02	0.95 [0.59-1.54]	0.846	1.06 [0.85-1.33]	0.581	0.85 [0.62-1.18]	0.329
IGL	1.22 [1-1.48]	5.04e-02	0.98 [0.61-1.56]	0.920	1.1 [0.86-1.41]	0.445	0.95 [0.73-1.24]	0.717
TRA	1.16 [0.84-1.61]	0.359	1.05 [0.45-2.42]	0.918	1.33 [0.91-1.95]	0.143	0.89 [0.62-1.27]	0.512
TRB	1.31 [0.93-1.85]	0.128	0.85 [0.3-2.38]	0.752	1.27 [0.87-1.85]	0.208	0.73 [0.47-1.12]	0.146
**Diversity**								
entropy								
IGH	1.14 [0.97-1.36]	0.120	1 [0.63-1.56]	0.986	1.18 [0.94-1.48]	0.156	1.03 [0.8-1.31]	0.841
IGK	1.42 [1.1-1.84]	7.39e-03	1.22 [0.75-2]	0.419	1.6 [1.07-2.39]	2.11e-02	1.42 [1.01-1.99]	4.10e-02
IGL	1.21 [0.94-1.56]	0.140	1.28 [0.71-2.28]	0.414	1.26 [0.89-1.78]	0.193	1.28 [0.96-1.7]	9.67e-02
TRA	1.11 [0.85-1.46]	0.430	1.22 [0.81-1.84]	0.347	1.33 [0.99-1.78]	5.71e-02	0.85 [0.64-1.12]	0.251
TRB	1.16 [0.87-1.54]	0.319	1.22 [0.81-1.85]	0.346	1.43 [1.06-1.92]	1.87e-02	1.07 [0.82-1.38]	0.631

Figure S1. Correlation between mutational rates and inflammatory score with diversity by subtypes. BCR related results are plotted in purple and TCR in yellow. In the Y axis the logarithm of the expression is displayed. On the X axes, the logarithm 10 of the A) non-silent B) silent mutational rates, C) inflammation score are plotted. Each line, is the regression line assessed in the correlation test performed by subtypes and they are colored by them.

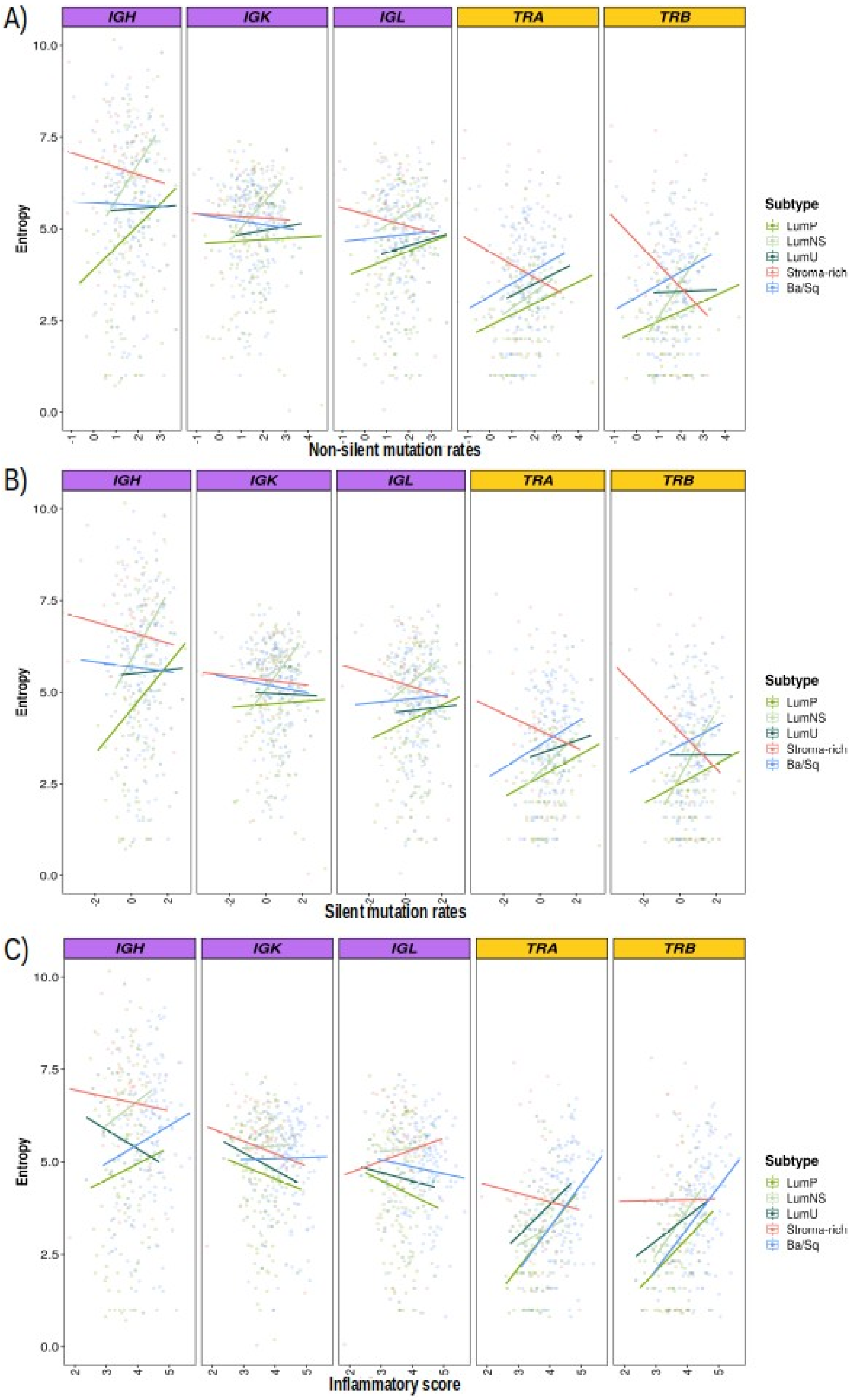

Supplement: Supplementary file 1 [file DataSheet_1.pdf]
